# Disparities in overall survival by varying duration of disability in activities of daily living in older people: A population-based cohort from Chinese Longitudinal Healthy Longevity Survey (CLHLS)

**DOI:** 10.1016/j.jnha.2023.100022

**Published:** 2024-01-01

**Authors:** Lu Liu, Yi Zheng, Jiawei Tian, Liying Li, Haiyan Ruan, Shanshan Jia, Xin Zhang, Runyu Ye, Xianghao Zuo, Xiaoping Chen, Sen He

**Affiliations:** a Department of Cardiology, West China Hospital, Sichuan University, Chengdu, China; b School of Public Health, Wuhan University, Wuhan, China; c Department of Cardiology, Hospital of Traditional Chinese Medicine, Shuangliu District, Chengdu, China

**Keywords:** Activities of daily living, Disability, Survival, Older people, Accelerated failure time

## Abstract

**Objectives:**

To investigate the association between duration of disability in activity of daily living (ADL) and overall survival in older individuals.

**Design:**

A prospective cohort study.

**Setting:**

Community-based data from Chinese Longitudinal Healthy Longevity Survey.

**Participants:**

In total, 13,560 participants without ADL disability and 2772 participants with ADL disability at baseline were included.

**Measurements:**

ADL disability was assessed using Katz index scale, which included six essential ADLs: dressing, bathing, transferring, toileting, continence, and eating. Dependence of each item was scored on a scale of 1, the maximum total score was 6. At baseline, duration of ADL disability was defined as the maximum duration among the six items. The study outcome was overall survival. Accelerated failure time models were constructed to investigate the association between duration of ADL disability and overall survival. Subgroup analyses by sex, age, and multimorbidites, as well as sensitive analyses were conducted.

**Results:**

During 81,868.7 person-years follow-up, 11,092 deaths were recorded. Overall, ADL disability was associated with lower overall survival compared to non-ADL disability. With duration of ADL disability extending, the overall survival strikingly dropped in the first 12 months, reaching its lowest point with adjusted time ratio (TR) at 0.66 (95%CI: 0.61−0.72, p < 0.001), then moderately grew until the 60th month, finally stayed constant thereafter. Participants with ADL scores of 1−3 had higher survival compared to those with scores of 4−6, and both groups followed a similar trend of varied survival to the whole cohort. Moreover, subgroup analyses and sensitivity analyses showed the robustness of these findings.

**Conclusions:**

Our findings first address a golden time window for the older individuals with ADL disability. More attention should be given to them, especially in the first 12 months since diagnosis, to reduce mortality and extend the lifespan.

## 1 Introduction

The remarkable extension of life expectancy stands out as one of the most important accomplishments of the 20th century [1]. With advancements in healthcare and technology, it is predicted that individuals born in the 21st century from countries with longer life expectancies will have the potential to live up to 100 years and beyond [1]. However, as people age, a considerable proportion of them inevitably face vulnerability, increased frailty, as well as disability [2]. These age-related conditions can limit their ability to carry out everyday activities that are essential for maintaining independence in the community. Prior studies have reported approximately 10% of community-dwelling people aged 75 and over developed new-onset activities of daily living (ADL) disability each year [3], and 36.2% of older people aged 65 and over who were independent in climbing stairs and walking a half mile at baseline finally lost mobility over the four-year follow-up [4].

Moreover, 14.02 million more older Chinese people will need care for disability by 2030 [5]. However, taking care of disabled older people is an incredibly complicated and strenuous task [6]. The ability to adequately care for disabled older individuals is not solely determined by individual efforts, but it is also highly influenced by the larger socio-economic context [7], such as the structure of healthcare systems, government policies, and social welfare programs, which are essential determinants of the resources and services available to support this vulnerable population. Therefore, it is crucial to gain a deeper understanding of the impact of ADL disability on the overall well-being and prognosis of older people in China.

Substantial evidence has demonstrated that disability in older people is strongly correlated with an increased risk of mortality [[8], [9], [10]]. These studies have primarily focused on examining the long-term association between disability and mortality, confirming disability as a significant risk factor. However, it is important to recognize that the impact of disability on survival may not be a homogeneous condition, which may change dynamically. In other words, it is crucial to understand whether the relationship between disability and subsequent outcomes remain constant or if it evolves over different stages of disability. To the authors’ knowledge, no previous study has investigated the associations. Thus, our study aimed to fill this crucial research gap by investigating the association between ADL disability duration and overall survival in older Chinese individuals, using the data from the Chinese Longitudinal Healthy Longevity Survey (CLHLS).

## 2 Methods

### 2.1 Study participants

The present analyses were based on CLHLS, a nationwide, ongoing, prospective cohort study of community-dwelling Chinese older people. In brief, the CLHLS was carried out in a randomly selected half of the counties and cities in 23 of the 31 provinces, accounting for approximately 85.0% of the total population of China. Launched in 1998, the survey carried out follow-ups every 2 or 3 years since then with subsequent follow-ups in 2000, 2002, 2005, 2008, 2011, 2014, and 2018. To account for the attrition due to death and loss of follow-up, new participants were enrolled during the following waves from 1998. The surveys were administered in participants' homes by trained interviewers with a structured questionnaire. Other details concerning the objectives, designs, and methods of the CLHLS can be found elsewhere [11,12], and data quality was reported to be generally good [13]. CLHLS was conducted in accordance with the principles outlined in the Declaration of Helsinki [14], and was approved by the Research Ethics Committee of Peking University (IRB00001052-13074). All participants or their proxy respondents provided written informed consent.

**Fig. 1:**
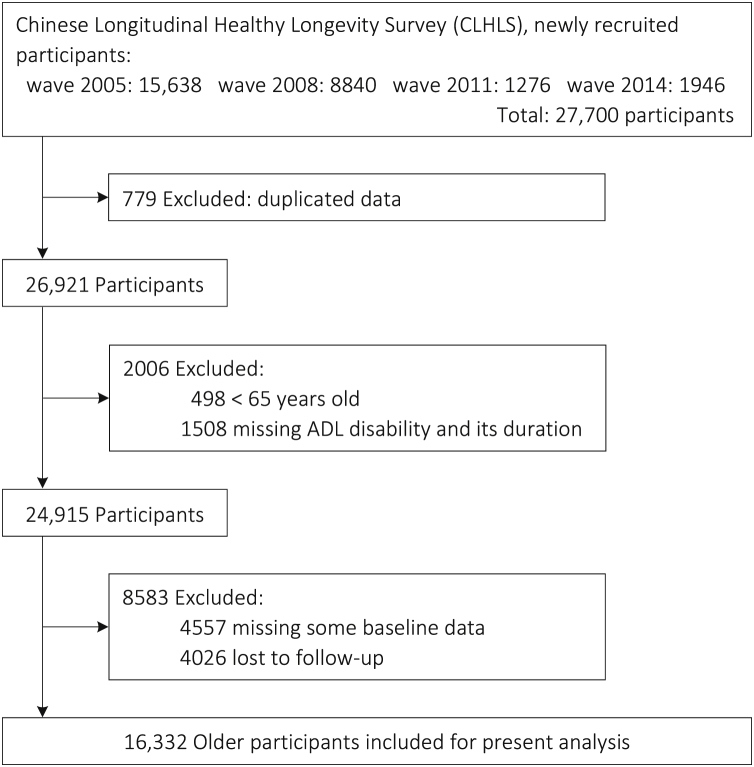
Flow chart of the study sample enrollment.

In order to collect information on the duration of ADL disability, the present study incorporated data from four consecutive waves that took place in 2005, 2008, 2011, and 2014. These waves were particularly chosen because they recorded the collection of data pertaining to the duration of ADL disability since 2005. The final interview was the 2018–2019 wave. Fig. 1 illustrates the flow chart of participants' enrollment. Finally, the analyzed sample comprised 16,332 older people aged ≥ 65 years.

### 2.2 Assessment of ADL disability

Disability is a measure of the extent to which an older person is incapable of performing the fundamental tasks without assistance [2]. In the CLHLS survey, ADL disability was assessed based on Katz index scale [15], which graded individuals on a scale of dependent or independent for the hierarchical ordering of 6 items, including dressing, bathing, transferring, toileting, continence, and eating. Each item included three answers: complete independence, partial dependence, and complete dependence. ADL disability was defined as present if participants needed any assistance in performing at least one of the six items [16]. For the participants with ADL disability, the duration of disability for each item was further assessed by the question “If receiving assistance, for how long?”; then, the duration of ADL disability for each participant was defined as the maximum duration among the six items. Finally, 13,560 participants were classified as non-ADL disability, and ADL disability was ascertained among 2772 participants whose score and duration distributions of ADL disability are shown in Figure S1A and 1B, respectively. Duration of ADL disability that lasted over 150 months were coded as a value of 150, due to most of durations less than 150 months (Figure S1B).

In the present study, dependence of each item was coded 1 score, so the maximum score was 6. Then, compared with non-ADL disability, we focused on four specific duration windows of ADL disability for analysis of overall survival: >0 and <6 consecutive months, ≥6 and <12 consecutive months, ≥12 and <60 consecutive months, and ≥60 months.

### 2.3 Assessment of other covariates

Table S1 depicts detailed information of other covariates, including sex, age, education, marital status, residence, co-residence, current smoking, current drinking, current regular exercise, regular intake of foods (fruits, vegetables, meats, fishes, eggs, and beans), multicomorbidities (hypertension, diabetes, heart diseases, cerebrovascular diseases, respiratory diseases, and cancer), and self-rated health, which were obtained through questionnaires. More detailed information about these covariates can be found at: https://agingcenter.duke.edu/CLHLS.

### 2.4 Study outcome

The study outcome was set as overall survival, defined as the time from baseline to any cause of death. During each survey, the survival status of the decedents was collected through close family members. All participants were followed up from the first evaluation up to death or the most recent evaluation. In addition, the reliability of mortality in the CLHLS may be more reliable than those derived from the national census, although some recall errors occurred [17,18].

### 2.5 Statistical analysis

Baseline characteristics of the study population were described as median (interquartile range, IQR) for the continuous variables or number (percentage) for the categorical variables. Comparisons of baseline characteristics were performed using Kruskal-Wallis tests for continuous variables, and the chi-square or Fisher exact tests for categorical variables.

The probability of overall survival was displayed by Kaplan-Meier plots, and comparisons were conducted by use of log-rank test. Owing to a violation of the proportional hazards assumption in the Cox proportional hazards models indicated by schoenfeld residuals, the parametric accelerated failure time (AFT) models were applied for the better goodness-of-fit of the model to the observed data and more robust statistical inference under such circumstances [19,20]. The AFT model estimates the time ratio (TR, sometimes called acceleration factor), which describes the estimated delay until an event occurs with treatment relative to the control group. In other words, TR estimated as α means that the lifespan of this group, on average, is stretched out α‑times longer than that of the reference group [21], thus, TR over 1 is equivalent to a lower risk. Several different distributions (Weibull, Exponential, Gaussian, Logistic, Lognormal, LogLogistic) with the Akaike Information Criterion (AIC) were compared, and a smaller value indicated the Weibull AFT model to be a better fit (Table S2). Meanwhile, we explored the dose-response relationship between duration of ADL disability and overall survival by adjusted smooth curve fitting using restricted cubic spline [22].

Moreover, we performed subgroup analyses by specific parameters: sex (male, female), age (< 100 years, ≥ 100 years), and multimorbidity (yes, no). In addition, to assess the robustness of the main findings, we performed a series of sensitivity analyses, including: [1] to reduce potential reverse causation between ADL disability and death, we excluded the deaths within the first year or the first two years of follow-up, in other words, it could be because in the last phase of the life that some older people had difficulties in ADL [2]; to clarify the role of participants lost to follow-up in the associations, we did sensitivity analyses assuming such participants censored at two time points: median or the end of follow-up [3]; Table S3 presents the distributions of baseline variables with missing data, we deleted the cases with missing data in the main analyses, with multiple imputation as another sensitivity analysis.

All analyses were performed with R version 4.1.0 including the “compareGroups”, “survival”, “tidyverse”, “rms”, “mice”, “forestplot”, “survminer”, and “stats” packages (http://www.R-project.org). All tests were two-sided, and p values <0.05 were considered statistically significant.

## 3 Results

### 3.1 Baseline characteristics

The baseline characteristics of the 16,332 participants are presented in Table 1. Participants were categorized by their baseline ADL disability status (no or yes), wherein participants suffering from ADL disability were further divided into four groups according to their duration of ADL disability at baseline. Out of the total participants, 7267 were male, accounting for 44.50% of the sample. The median age of the included participants was 87.00 years. On the whole, female participants were more susceptible to ADL disability. Current smokers, current drinkers, and the participants engaging in regular exercise had a larger proportion of non-ADL disability, which may be attributed to easier access to alcohol, cigarette, and exercise for those without ADL disability. As for multimorbidities, the non-ADL disability group had the least patients with diabetes, heart diseases, cerebrovascular diseases, and respiratory diseases, meanwhile, they were more inclined to evaluate their own health status as good.

**Table 1:** Baseline characteristics of the included participants.

Variable	All	Non-ADL disability	Duration of ADL disability				p for trend^a^
			Less than 6 months	6–12 months	12–60 months	At least 60 months	
No. of participants	16,332	13,560	530	350	1442	450	
Sex: male	7267 (44.50%)	6399 (47.19%)	179 (33.77%)	119 (34.00%)	461 (31.97%)	109 (24.22%)	<0.001
Age (years)	87.00 (77.00−95.00)	85.00 (74.00−92.00)	94.00 (87.00−100.00)	94.00 (88.25−100.00)	97.00 (90.00−101.00)	100.00 (91.00−101.00)	<0.001
Education							<0.001
No school	10,146 (62.12%)	8118 (59.87%)	392 (73.96%)	244 (69.71%)	1052 (72.95%)	340 (75.56%)	
1 year or more	6186 (37.88%)	5442 (40.13%)	138 (26.04%)	106 (30.29%)	390 (27.05%)	110 (24.44%)	
Marital status							<0.001
Not in marriage	10,634 (65.11%)	8289 (61.13%)	426 (80.38%)	307 (87.71%)	1218 (84.47%)	394 (87.56%)	
In marriage	5698 (34.89%)	5271 (38.87%)	104 (19.62%)	43 (12.29%)	224 (15.53%)	56 (12.44%)	
Residence							<0.001
Urban	6174 (37.80%)	4887 (36.04%)	227 (42.83%)	167 (47.71%)	660 (45.77%)	233 (51.78%)	
Rural	10,158 (62.20%)	8673 (63.96%)	303 (57.17%)	183 (52.29%)	782 (54.23%)	217 (48.22%)	
Co-residence							<0.001
With family members	13,493 (82.62%)	11,008 (81.18%)	451 (85.09%)	313 (89.43%)	1301 (90.22%)	420 (93.33%)	
Alone	2534 (15.52%)	2327 (17.16%)	63 (11.89%)	26 (7.43%)	93 (6.45%)	25 (5.56%)	
In an institution	305 (1.87%)	225 (1.66%)	16 (3.02%)	11 (3.14%)	48 (3.33%)	5 (1.11%)	
Current smoking	3206 (19.63%)	2937 (21.66%)	50 (9.43%)	34 (9.71%)	145 (10.06%)	40 (8.89%)	<0.001
Current drinking	3333 (20.41%)	2944 (21.71%)	67 (12.64%)	49 (14.00%)	199 (13.80%)	74 (16.44%)	<0.001
Current regular exercise	4781 (29.27%)	4259 (31.41%)	90 (16.98%)	59 (16.86%)	271 (18.79%)	102 (22.67%)	<0.001
Regular intake of foods							
Fruits	6194 (37.93%)	5042 (37.18%)	177 (33.40%)	131 (37.43%)	606 (42.02%)	238 (52.89%)	<0.001
Vegetables	14,246 (87.23%)	11,966 (88.24%)	430 (81.13%)	287 (82.00%)	1184 (82.11%)	379 (84.22%)	<0.001
Meats	8383 (51.33%)	7014 (51.73%)	233 (43.96%)	175 (50.00%)	699 (48.47%)	262 (58.22%)	0.486
Fishes	5088 (31.15%)	4235 (31.23%)	141 (26.60%)	90 (25.71%)	447 (31.00%)	175 (38.89%)	0.268
Eggs	9026 (55.27%)	7299 (53.83%)	271 (51.13%)	190 (54.29%)	932 (64.63%)	334 (74.22%)	<0.001
Beans	7565 (46.32%)	6162 (45.44%)	247 (46.60%)	164 (46.86%)	750 (52.01%)	242 (53.78%)	<0.001
Multimobidities							
Hypertension	3115 (19.07%)	2566 (18.92%)	106 (20.00%)	72 (20.57%)	290 (20.11%)	81 (18.00%)	0.447
Diabetes	372 (2.28%)	288 (2.12%)	15 (2.83%)	8 (2.29%)	47 (3.26%)	14 (3.11%)	0.003
Heart diseases	1330 (8.14%)	997 (7.35%)	46 (8.68%)	36 (10.29%)	192 (13.31%)	59 (13.11%)	<0.001
Cerebrovascular diseases	789 (4.83%)	520 (3.83%)	47 (8.87%)	34 (9.71%)	148 (10.26%)	40 (8.89%)	<0.001
Respiratory diseases	1814 (11.11%)	1468 (10.83%)	71 (13.40%)	42 (12.00%)	167 (11.58%)	66 (14.67%)	0.017
Cancer	75 (0.46%)	51 (0.38%)	10 (1.89%)	4 (1.14%)	7 (0.49%)	3 (0.67%)	0.063
Self-rated health							<0.001
Good	8540 (52.29%)	7374 (54.38%)	162 (30.57%)	121 (34.57%)	656 (45.49%)	227 (50.44%)	
Fair	5393 (33.02%)	4487 (33.09%)	187 (35.28%)	118 (33.71%)	463 (32.11%)	138 (30.67%)	
Poor	2399 (14.69%)	1699 (12.53%)	181 (34.15%)	111 (31.71%)	323 (22.40%)	85 (18.89%)	

Abbreviations: ADL = activities of daily living, IQR = inter-quartile range.

Values are median (IQR) or n (%).

a For these groups: non-ADL disability, less than 6 months, 6–12 months, 12–60 months, and at least 60 months.

### 3.2 Association between duration of ADL disability and overall survival

**Fig. 2:**
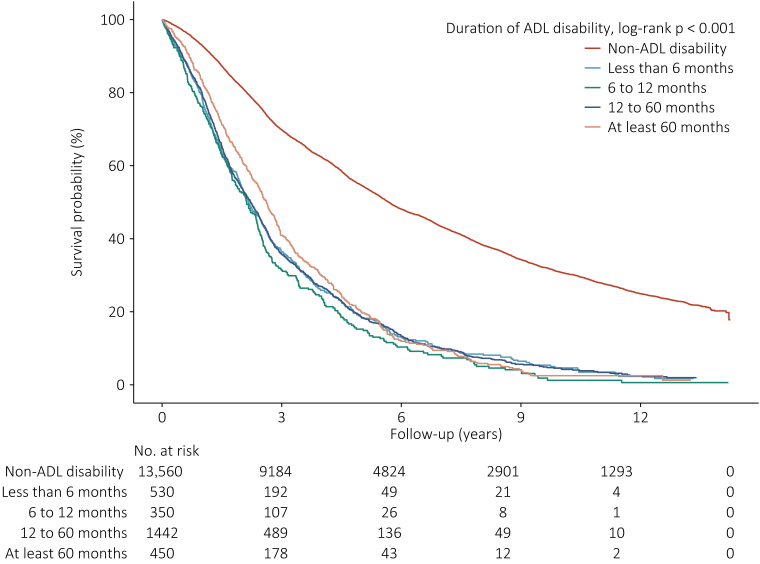
Kaplan-Meier curves plotting survival probability of non-ADL disability participants and those in different duration of ADL disability during follow-up.

**Fig. 3:**
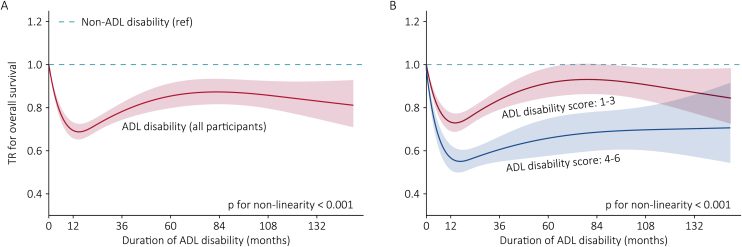
Dose-response relationship between duration of ADL disability and overall survival. *Note:* With non-ADL disability as reference, the red and blue solid lines depict the trend in overall survival with longer duration of ADL disability, while shadow represents corresponding 95% CIs (A for all participants, B for participants with different ADL disability score). Duration of ADL disability was modeled using a restricted cubic spline with four knots at the 5th, 35th, 65th, and 95th percentiles, and TRs and 95% CIs were derived from AFT model adjusted for sex, age, education, marital status, residence, co-residence, current smoking, current drinking, current regular exercise, regular intake of foods, comorbidities, and self-rated health. Abbreviations: ADL = activities of daily living, AFT = accelerated failure time, CI = confidence interval, TR = time ratio

During a period of 81,868.7 person-years of follow-up, a total of 11,092 deaths were documented. The mortality rates for non-ADL disability participants were 11.6 (95%CI: 11.4−11.9) per 100 person-years. However, for participants in different duration of ADL disability, the mortality rates were 31.6 (95%CI: 29.2−34.0), 35.7 (95%CI: 32.5−38.8), 31.6 (95%CI: 30.2−33.1), and 30.1 (95%CI: 27.6−32.5) per 100 person-years, respectively. Accordingly, Kaplan-Meier curves also demonstrated that the survival probability was significantly higher in non-ADL disability (log-rank p < 0.001) (Fig. 2). Table 2 shows the association between duration of ADL disability and overall survival. With non-ADL disability as a reference, the adjusted TRs were 0.73 (95%CI: 0.68−0.79), 0.66 (95%CI: 0.61−0.72), 0.76 (95%CI: 0.73−0.80), and 0.82 (95%CI: 0.76−0.88) for different duration, respectively. Adjusted TRs for overall survival in ADL disability in all time windows were below 1, indicating ADL disability had a significant accelerating effect on lifespan, and the lowest TR (0.66) was in the 12th-60th months, which meant it took 0.66-folds longer of time relative to non-ADL disability group to reach death. Fig. 3A visualized the association, TR for overall survival declined dramatically in the first 12 months with a nadir at the 12th month, and then rose slowly until the 60th month with a plateau afterward; however, the overall survival never reached the level seen in non-ADL disability group.

**Table 2:** Association between duration of ADL disability and overall survival.

	Non-ADL disability	Duration of ADL disability			
		Less than 6 months	6–12 months	12–60 months	At least 60 months
• *All participants*					
No. of participants	13,560	530	350	1442	450
Deaths (n)	8614	470	319	1278	411
Follow-up (PYs)	74,080.5	1487.6	894.7	4038.8	1367.1
Mortality rate (95% CI)^a^	11.6 (11.4−11.9)	31.6 (29.2−34.0)	35.7 (32.5−38.8)	31.6 (30.2−33.1)	30.1 (27.6−32.5)
Unadjusted TR (95% CI)	1.00 (ref)	0.38 (0.35−0.42), <0.001	0.34 (0.31−0.38), <0.001	0.38 (0.36−0.41), <0.001	0.40 (0.37−0.44), <0.001
Adjusted TR (95% CI)					
model 1^b^	1.00 (ref)	0.68 (0.63−0.73), <0.001	0.62 (0.56−0.67), <0.001	0.74 (0.70−0.77), <0.001	0.81 (0.75−0.87), <0.001
model 2^c^	1.00 (ref)	0.73 (0.68−0.79), <0.001	0.66 (0.61−0.72), <0.001	0.76 (0.73−0.80), <0.001	0.82 (0.76−0.88), <0.001

• *ADL disability score: 1−3*					
No. of participants	13,560	406	259	1122	349
Deaths (n)	8614	352	233	982	313
Follow-up (PYs)	74,080.5	1229.6	708.9	3443.9	1154.9
Mortality rate (95% CI)^a^	11.6 (11.4−11.9)	28.6 (26.1−31.2)	32.9 (29.4−36.3)	28.5 (27.0−30.0)	27.1 (24.5−29.7)
Unadjusted TR (95% CI)	1.00 (ref)	0.42 (0.39−0.47), <0.001	0.37 (0.33−0.42), <0.001	0.42 (0.40−0.45), <0.001	0.45 (0.40−0.49), <0.001
Adjusted TR (95% CI)					
model 1^b^	1.00 (ref)	0.72 (0.67−0.78), <0.001	0.68 (0.61−0.75), <0.001	0.80 (0.76−0.84), <0.001	0.88 (0.81−0.96), 0.003
model 2^c^	1.00 (ref)	0.77 (0.71−0.83), <0.001	0.71 (0.65−0.78), <0.001	0.81 (0.77−0.85), <0.001	0.88 (0.81−0.96), 0.003

• *ADL disability score: 4−6*					
No. of participants	13,560	124	91	320	101
Deaths (n)	8614	118	86	296	98
Follow-up (PYs)	74,080.5	257.9	185.8	594.9	212.2
Mortality rate (95% CI)^a^	11.6 (11.4−11.9)	45.7 (39.7−51.8)	46.3 (39.1−53.5)	49.8 (45.7−53.8)	46.2 (39.5−52.9)
Unadjusted TR (95% CI)	1.00 (ref)	0.27 (0.23−0.31), <0.001	0.27 (0.22−0.32), <0.001	0.24 (0.22−0.27), <0.001	0.26 (0.22−0.32), <0.001
Adjusted TR (95% CI)					
model 1^b^	1.00 (ref)	0.56 (0.49−0.64), <0.001	0.46 (0.39−0.54), <0.001	0.56 (0.51−0.61), <0.001	0.60 (0.52−0.70), <0.001
model 2^c^	1.00 (ref)	0.63 (0.55−0.72), <0.001	0.52 (0.45−0.62), <0.001	0.62 (0.57−0.68), <0.001	0.65 (0.56−0.75), <0.001

Abbreviations: ADL = activities of daily living, CI = confidence interval, PYs = person-years, TR = time ratio.

a per 100 PYs.

b model 1 with adjustment for sex and age.

c model 2 with adjustment for model 1 plus other covariates, including education, marital status, residence, co-residence, current smoking, current drinking, current regular exercise, regular intake of foods, multimorbidities, and self-rated health.

When ADL disability was categorized by severity (scoring 1–6), significant differences in overall survival could be observed in comparison with non-ADL disability (Table S4 and Figure S2), and there was a downward trend in survival probability along with an upward trend in cumulative mortality rate with the score increasing. Given this, we visualized TRs for overall survival of the six severity levels respectively (Figure S3). The participants scored 1–3 could reach the survival equivalent to those without ADL disability before 60 months, as indicated by the 95% confidence interval crossing 1, whereas the participants scored 4–6 were less likely to live long as the non-ADL disability group. Therefore, we reclassified the included participants into three groups for further analysis: non-ADL disability, ADL disability scored 1−3, and ADL disability scored 4−6.

With non-ADL disability as a reference, the varied association between duration of ADL disability and overall survival of the two ADL disability groups were presented in Fig. 3B. Similar to the entire cohort (Fig. 3A), the extents of time acceleration for lifespan of the two ADL disability groups were both gradually enhanced with time in the first 12 months, then slowly attenuated during the 12th-60th months with the TR remaining below 1, and dynamically balanced afterward. Table 2 shows the detailed results, in comparison with non-ADL disability, ADL disability in each time course was associated with a lower adjusted TR. Wherein, in participants with a score of 1−3, the 6th–12th months was associated with the minimum adjusted TR at 0.71 (95%CI: 0.65−0.78), while the final time section (60 months and over) was associated with the maximum adjusted TR at 0.88 (95%CI: 0.81−0.96). The same was true of the trend in participants with a score of 4−6 that the 6th–12th months was associated with the minimum adjusted TR at 0.52 (95%CI: 0.45−0.62), while the final time section was associated with the maximum adjusted TR at 0.65 (95%CI: 0.56−0.75).

### 3.3 Subgroup analysis

A series of subgroup analysis by sex, age, and multimorbidities were conducted. As Table S5 displays, in male participants, the first 6 months was associated with the lowest survival (adjusted TR: 0.72, 95%CI: 0.64−0.81). In female participants, the lowest survival was observed during the 12th -60th months with adjusted TR at 0.59 (95%CI: 0.53−0.66). In participants younger than 100 years (Table S6), survival dynamically balanced at a lower level with adjusted TRs keeping at 0.48 in the first 60 months. In participants aged 100 years and over, all the adjusted TRs were estimated above 0.80 except for that of the 12th–60th months (adjusted TR: 0.67, 95%CI: 0.57−0.78). In participants without multicomorbidities (Table S7), the 12th -60th months remained to be associated with the lowest survival (adjusted TR: 0.65, 95%CI: 0.59−0.72), while in those with multcomorbidies, the lowest survival was observed in the first 6 months (adjusted TR: 0.66, 95%CI: 0.59−0.74).

### 3.4 Sensitivity analyses

Table S8-S10 displayed the results of a series of sensitivity analysis, which enhanced our findings in main analysis. After excluding the deceased persons within the first year or first two years (Table S8), the lowest TRs of the whole cohort were both observed in the 12th-60th months, i.e, 0.73 (95%CI: 0.67−0.79) and 0.77 (95%CI: 0.71−0.83), respectively. In addition, when the participants lost to follow-up (Table S9) were assumed to censor at the median of follow-up, the 12th-60th months was associated with the lowest TR (0.74. 95%CI: 0.68−0.80) for the whole cohort. Similar results could be observed when they were assumed to censor at the end of follow-up with the TR estimated as 0.82 (95%CI: 0.72−0.93) for the 12th-60th months. Finally, multiple imputation was used to handle the cases with missing baseline data (Table S10). On account of the partial overlap between the lost participants and the participants with missing baseline data, the present sample size (n = 20,014) was a little different from the sample size (n = 20,889) in the flow chart (Fig. 1). The 12th–60th months remained to be associated with the lowest TRs, namely, 0.67 (95%CI: 0.62−0.72) for the whole cohort.

## 4 Discussion

To the authors’ knowledge, this is a pioneering study to investigate the association between duration of ADL disability and overall survival in older people by use of AFT models. The highlight of our research is that throughout the entire course of ADL disability, overall survival varies over time with the lowest survival always occurring in the first 12 months, and in this time window, survival constantly declined with time. Our findings first address a golden time window for the older individuals with ADL disability. More attention should be given to them, especially in the first 12 months, to reduce mortality and extend the lifespan.

In the present study, there are something worth discussing as well. Firstly, as Table 1 shows, it seems the older participants with risk behaviors (current smoking and drinking) had a larger proportion of non-ADL disability. On one hand, the understanding of older participants regarding the assistance to performing basic daily activities was subjective, resulting in ADL disability imperceptible to a proportion of participants, who were then classified as non-ADL disability according to their responses to the questionnaire. On the other hand, there may be potential reverse causation. Specifically, “current smoking” was determined by “Do you smoke at present?”, therefore, the participants with smoking history but having quitted smoking before baseline were not included, among them, however, there were probably a considerable proportion of participants quitting smoking for health-related problems, like ADL disability. Thus, the participants continued to smoke were less likely to have trouble with ADL. Secondly, in Figure S3B-3D, where the 95% confidence interval crossed the reference line at some later time points indicated an equivalent survival to non-ADL disability group. For the older people scored 2 (Figure S3B), it was because of the relatively milder ADL disability condition that the survival can gradually rise after the 12th month, and finally at an earlier time point (the 60th month) reach the level of non-ADL disability. For those scored 3 (Figure S3C), after the 12th month, the survival gradually rose, then also reached the level of non-ADL disability at around the 60th month. From then on, the impact of ADL disability on long-term prognosis, i.e, survival, showed slightly negative with duration. For those scored 4 (Figure S3D), the survival gradually rose at a lower speed until the 108th month (9 years), however, the time might be exactly human’s end of natural lifespan. In other words, before the negative association at the later time points (like that in Fig. 3C) can be observed in those scoring 4 (Figure S3D), the older people has already reached the final stage of the life. Thirdly, when the participants were grouped by age in Table S6, it seemed that the overall survival of the participants with ADL disability relative to non-ADL disability was higher in those aged 100 and more rather than in the younger group. It could be attributed to that in non-ADL disability people, the physiological natural mortality was higher in people aged over 100 years than those aged < 100. This means that regardless of ADL disability, centenarians were already nearing the end of their lives. It was true that as age increased, the gap in life expectancy between disability-free and ADL disability got shorter [23].

Why overall survival of the older individuals with ADL disability decreased so strikingly in the first 12 months? Future studies are warranted to explore the mechanisms. There are several potential reasons. Firstly, ADL disability can be directly caused by stroke and dementia [24,25], in the case of older individuals newly diagnosed with ADL disability, primary diseases are usually in an acute stage, which may lead to a high mortality. Secondly, ADL disability is one complication of several major noncommunicable diseases, such as hypertension, cancers, chronic respiratory diseases, and diabetes [[26], [27], [28], [29]]. Diabetes can accelerate the occurrence of ADL disability by 6−11 years during aging [30,31]. Hypertension was also associated with 91% higher risk for incident or worsening ADL disability (OR: 1.91, 95% CI: 1.06–3.43) [32]. The incidence of ADL disability reflects the aggravation of the primary diseases. Thirdly, when older people are diagnosed with disability and even have to be bedridden, a psychological gap develops [33]. Thus, they think that they can no longer retain dignity and have a poor quality of life. Such is the case, disability has been confirmed to be associated with depression in older people [[34], [35], [36]]. Disability significantly contributed to the onset of depressive symptoms and vice versa [35]. A multi-center cohort study consisting of 6124 Chinese aged ≥ 60 years reported that participants with depression subcase and case had 46% and 45% increased 10-year risk of mortality, respectively [37].

Timely interventions for disability, primary diseases, as well as mental disorders may be warranted, in particular in the first 12 months since ADL disability diagnosis. By implementing these multidimensional interventions concurrently, older individuals with ADL disability can benefit quantity of lives. Simultaneously, the quality of life can be significantly enhanced as these interventions can alleviate symptoms, improve functional abilities, promote emotional well-being, and enhance overall satisfaction with life.

Last but not least, it is important to acknowledge several limitations in the present study. Firstly, we focused on the baseline condition of ADL disability and subsequent overall survival, in fact, there are sometimes trajectories of ADL disability, which involve changes in overall survival. Based on the present analyses (Figure S3), if disability improves, the likelihood of overall survival may increase, but caution should be kept in mind as well if it is still in the first 12 months, after all, ADL disability with a score 1 in this time window is associated with the lowest survival; if disability progresses, at least the next 12 months should be carefully supervised whatever time sections it is before. Further studies are required to explore the association between trajectories of ADL disability and overall survival. Secondly, the cohort only consists of Chinese, whether the similar findings can be extrapolated to different ethnicities and distributions is unknown. Thirdly, due to certain information collection by self-report and questionnaires in CLHLS, recall bias could be resulted in.

## 5 Conclusion

ADL disability was associated with a significantly decreased overall survival in older people. During ADL disability, overall survival varied over time, which dropped rapidly with a nadir in the 12th month, then rose slowly until the 60th month, and finally dynamically balanced. Our findings first address a golden time window for the older individuals with ADL disability. More attention should be given to them, especially in the first 12 months since diagnosis, to reduce mortality and extend the lifespan.

## Funding

The study was supported by Sichuan Science and Technology Program, China (Grant No. 2022YFS0186), the National Natural Science Foundation of China (Grant No. 81600299), and the National Natural Science Foundation of China (Grant No. 81970355).

## Ethics approval and consent to participate

The CLHLS study was approved by the Research Ethics Committee of Peking University (IRB00001052-13074), and all participants or their proxy respondents provided written informed consent.

## Declaration of competing interest

The authors have declared no conflict of interest.

## Availability of data and materials

The CLHLS questionnaires are available at https://sites.duke.edu/centerforaging/programs/chinese-longitudinal-healthy-longevity-survey-clhls/survey-documentation/questionnaires/. The full datasets used in this analysis are available from the corresponding author upon reasonable request.
