# Supplementary material for: Disparities in overall survival by varying duration of disability in activities of daily living in older people: A population-based cohort from Chinese Longitudinal Healthy Longevity Survey (CLHLS)
Source: J Nutr Health Aging. 2024 Jan 1;28(1):100022. doi: 10.1016/j.jnha.2023.100022 (PMC12877755; doi:10.1016/j.jnha.2023.100022)
Supplement: Supplementary file 1 [file mmc1.docx]

**Supplementary Materials**

**Disparities in overall survival by varying duration of disability in activities of daily living in older people: a population-based cohort from** **Chinese Longitudinal Healthy Longevity Survey (****CLHLS)**

Lu Liu Ph.D ^1, †^, Yi Zheng M.D ^1, †^, Jiawei Tian M.S ^2^, Liying Li M.S ^1^, Haiyan Ruan M.S ^1, 3^, Shanshan Jia Ph.D ^1^, Xin Zhang M.D ^1^, Runyu Ye M.D ^1^, Xianghao Zuo M.S ^1^, Xiaoping Chen M.D ^1, *^, Sen He M.D, Ph.D ^1, *^

^1^ Department of Cardiology, West China Hospital, Sichuan University, Chengdu, China.

^2^ School of Public Health, Wuhan University, Wuhan, China.

^3^ Department of Cardiology, Hospital of Traditional Chinese Medicine, Shuangliu District, Chengdu, China.

^*^ Corresponding author: Xiaoping Chen and Sen He; Department of Cardiology, West China Hospital, Sichuan University, No.37 Guoxue Alley, Chengdu, China; Email: xiaopingchen0196@163.com (for Xiaoping Chen), [hesensubmit@163.com](mailto:hesensubmit@163.com) (for Sen He).

^†^ Lu Liu and Yi Zheng equally contributed to the article.

**Supplementary tables**

Table S1. Definitions of baseline variables in the present study

Table S2. Values of Akaike Information Criterion statistic of different distributions for survival time

Table S3. Distributions of baseline variables with missing data

Table S4. Association between ADL disability score and overall survival

Table S5. Association between duration of ADL disability and overall survival by sex

Table S6. Association between duration of ADL disability and overall survival by age

Table S7. Association between duration of ADL disability and overall survival by multimorbidities

Table S8. Association between duration of ADL disability and overall survival after excluding deaths within the first year or the first two years

Table S9. Association between duration of ADL disability and overall survival, in considering the losses censored at varying time of follow-up

Table S10. Association between duration of ADL disability and overall survival after multiple imputation

**Supplementary figures**

Figure S1. The distributions of (A) ADL disability score and (B) duration of ADL disability in participants suffering from ADL disability at baseline.

Figure S2. Kaplan-Meier curves plotting survival probability of non-ADL disability participants and those with ADL disability in different severity.

Figure S3. Visualized association between duration of ADL disability and survival in participants with different severity of ADL disability.

| Table S1. Definitions of baseline variables in the present study | | | | |
| --- | --- | --- | --- | --- |
| Variable | Components of variable | Questions in the CLHLS questionnaire | Options in the questionnaire | Reclassification in the present study |
| Sex |  |  | • male • female | • Male • Female |
| Age |  |  |  | • Continuous (years) |
| Education |  | How many years did you attend school? | • years of school • don't know • missing | • No school: years of school = 0 • 1 year or more: years of school ≥ 1 • missing: don't know, missing |
| Marital status |  | Current marital status? | • currently married and living with spouse • separated • divorced • widowed • never married • don't know • missing | • In marriage: currently married and living with spouse, separated • Not in marriage: divorced, widowed, never married • missing: don't know, missing |
| Residence |  | Current residence area of interviewee? | • city • town  • rural | • Urban: city, town • Rural: rural |
| Co-residence |  | Co-residence? | • with household member(s) • alone • in an institution • missing | • With household member(s) • Alone • In an institution • missing |
| Current smoking |  | Do you smoke at present? | • yes • no • missing | • Current smoking: yes • No smoking at present: no • missing |
| Current drinking |  | Do you drink at present? | • yes • no • don't know • missing | • Current drinking: yes • No drinking at present: no • missing: don't know, missing |
| Current regular exercise |  | Do you do exercises regularly at present? | • yes • no • don't know • missing | • Current regular exercise: yes • No regular exercise at present: no • missing: don't know, missing |
| Regular intake of fruits, and vegetables, respectively |  | Do you eat these foods, respectively? | • almost everyday • except winter/quite often • occasionally • rarely or never • don't know • missing | • Regular intake: almost everyday, except winter/quite often • No regular intake: occasionally, rarely or never • missing: don't know, missing |
| Regular intake of meats, fishes, eggs, and beans, respectively |  | Do you eat these foods, respectively? | Wave 2005 • almost everyday • occasionally • rarely or never • missing  Waves 2008, 2011, 2014 • almost everyday • not everyday, but at least once per week • not every week, but at least once per month • not every month, but occasionally • rarely or never • don't know • missing | • Regular intake: almost everyday; not everyday, but at least once per week • No regular intake: not every week, but at least once per month; not every month, but occasionally; occasionally; rarely or never. • missing: don't know, missing |
| Hypertension, diabetes, heart diseases, cerebrovascular diseases, respiratory diseases, and cancer, respectively |  | Are you suffering from these diseases, respectively? | • yes • no • don't know • missing | • Yes: yes • No: no • missing: don't know, missing |
| Self-rated health |  | How do you rate your health at present? | • very good • good • so so • bad • very bad • not able to answer • missing | • Good: very good, good • Fair: so so • Poor: bad, very bad • missing: not able to answer, missing |
| More detailed information about these covariates can be found on: https://agingcenter.duke.edu/CLHLS. Abbreviations: ADL = activities of daily living, CLHLS = Chinese Longitudinal Healthy Longevity Surveys. | | | | |

| Table S2. Values of Akaike Information Criterion statistic of different distributions for survival time | |
| --- | --- |
| Distribution | Value of Akaike Information Criterion statistic |
| Weibull | 58403.0 |
| Exponential | 59684.0 |
| Gaussian | 66521.4 |
| Logistic | 66580.0 |
| Lognormal | 59684.2 |
| LogLogistic | 58852.6 |

Table S3. Distributions of baseline variables with missing data

| Variable | Number of missing data | Percentage of missing data (%) |
| --- | --- | --- |
| Sex | 0 | 0.00 |
| Age (years) | 0 | 0.00 |
| Education | 140 | 0.56 |
| Marital status | 19 | 0.08 |
| Residence | 0 | 0.00 |
| Co-residence | 29 | 0.12 |
| Current smoking | 12 | 0.05 |
| Current drinking | 20 | 0.08 |
| Current regular exercise | 50 | 0.20 |
| Regular intake of fruits | 4 | 0.02 |
| Regular intake of vegetables | 11 | 0.04 |
| Regular intake of meats | 9 | 0.04 |
| Regular intake of fishes | 8 | 0.03 |
| Regular intake of eggs | 9 | 0.04 |
| Regular intake of beans | 9 | 0.04 |
| Hypertension | 1044 | 4.19 |
| Diabetes | 1149 | 4.61 |
| Heart diseases | 1074 | 4.31 |
| Cerebrovascular diseases | 1096 | 4.40 |
| Respiratory diseases | 1017 | 4.08 |
| Cancer | 1318 | 5.29 |
| Self-rated health | 2448 | 9.83 |

Participants with missing values were deleted in the main statistical analyses, here we performed multiple imputation for missing values as a sensitivity analysis.

| Table S4. Association between ADL disability score and overall survival | | | | | | | |
| --- | --- | --- | --- | --- | --- | --- | --- |
|  | Non-ADL disability | ADL disability score | | | | | |
|  |  | 1 | 2 | 3 | 4 | 5 | 6 |
| No. of participants | 13560 | 1430 | 426 | 280 | 318 | 91 | 227 |
| Deaths (n) | 8614 | 1231 | 389 | 260 | 301 | 82 | 215 |
| Follow-up (PYs) | 74080.5 | 4672.8 | 1141.1 | 723.6 | 712.1 | 151.2 | 387.5 |
| Mortality rate (95% CI)^a^ | 11.6 (11.4-11.9) | 26.3 (25.1-27.6) | 34.1 (31.3-36.8) | 35.9 (32.4-39.4) | 42.3 (38.6-45.9) | 54.2 (46.3-62.2) | 55.5 (50.5-60.4) |
| Unadjusted TR (95% CI) | 1.00 (ref) | 0.46 (0.44-0.48), <0.001 | 0.36 (0.33-0.39), <0.001 | 0.34 (0.31-0.38), <0.001 | 0.29 (0.26-0.32), <0.001 | 0.22 (0.18-0.27), <0.001 | 0.22 (0.20-0.25), <0.001 |
| Adjusted TR (95% CI) |  |  |  |  |  |  |  |
| model 1^b^ | 1.00 (ref) | 0.80 (0.77-0.84), <0.001 | 0.76 (0.70-0.82), <0.001 | 0.64 (0.59-0.71), <0.001 | 0.59 (0.54-0.65), <0.001 | 0.45 (0.38-0.53), <0.001 | 0.50 (0.45-0.55), <0.001 |
| model 2^c^ | 1.00 (ref) | 0.81 (0.78-0.85), <0.001 | 0.79 (0.73-0.85), <0.001 | 0.69 (0.63-0.76), <0.001 | 0.64 (0.59-0.70), <0.001 | 0.49 (0.41-0.57), <0.001 | 0.57 (0.51-0.63), <0.001 |
| ^a^ per 100 PYs.  ^b^ model 1 with adjustment for sex and age.  ^c^ model 2 with adjustment for model 1 plus other covariates, including education, marital status, residence, co-residence, current smoking, current drinking, current regular exercise, regular intake of foods, multimorbidities, and self-rated health.  Abbreviations: ADL = activities of daily living, CI = confidence interval, PYs = person-years, TR = time ratio. | | | | | | | |

| Table S5. Association between duration of ADL disability and overall survival by sex | | | | | |
| --- | --- | --- | --- | --- | --- |
| Adjusted TR (95% CI)^a^, p | Non-ADL disability | Duration of ADL disability | | | |
|  |  | Less than 6 months | 6 to 12 months | 12 to 60 months | At least 60 months |
| Male |  |  |  |  |  |
| All participants | 1.00 (ref) | 0.72 (0.64-0.81), <0.001 | 0.78 (0.68-0.90), 0.001 | 0.73 (0.67-0.79), <0.001 | 0.81 (0.70-0.95), 0.008 |
| ADL disability score: 1-3 | 1.00 (ref) | 0.76 (0.67-0.86), <0.001 | 0.84 (0.72-0.99), 0.039 | 0.78 (0.72-0.85), <0.001 | 0.82 (0.70-0.97), 0.022 |
| ADL disability score: 4-6 | 1.00 (ref) | 0.58 (0.44-0.75), <0.001 | 0.54 (0.40-0.73), <0.001 | 0.56 (0.48-0.66), <0.001 | 0.81 (0.58-1.12), 0.201 |
| Female |  |  |  |  |  |
| All participants | 1.00 (ref) | 0.73 (0.67-0.80), <0.001 | 0.59 (0.53-0.66), <0.001 | 0.78 (0.73-0.82), <0.001 | 0.83 (0.76-0.91), <0.001 |
| ADL disability score: 1-3 | 1.00 (ref) | 0.77 (0.70-0.86), <0.001 | 0.62 (0.55-0.70), <0.001 | 0.82 (0.77-0.87), <0.001 | 0.90 (0.82-1.00), 0.052 |
| ADL disability score: 4-6 | 1.00 (ref) | 0.63 (0.54-0.74), <0.001 | 0.52 (0.43-0.62), <0.001 | 0.64 (0.57-0.71), <0.001 | 0.60 (0.51-0.72), <0.001 |
| ^a^ with adjustment for age, education, marital status, residence, co-residence, current smoking, current drinking, current regular exercise, regular intake of foods, multimorbidities, and self-rated health.  Abbreviations: ADL = activities of daily living, CI = confidence interval, TR = time ratio. | | | | | |

| Table S6. Association between duration of ADL disability and overall survival by age | | | | | |
| --- | --- | --- | --- | --- | --- |
| Adjusted TR (95% CI)^a^, p | Non-ADL disability | Duration of ADL disability | | | |
|  |  | Less than 6 months | 6 to 12 months | 12 to 60 months | At least 60 months |
| Age < 100 years |  |  |  |  |  |
| All participants | 1.00 (ref) | 0.48 (0.44-0.53), <0.001 | 0.48 (0.43-0.54), <0.001 | 0.48 (0.45-0.52), <0.001 | 0.52 (0.46-0.59), <0.001 |
| ADL disability score: 1-3 | 1.00 (ref) | 0.51 (0.46-0.57), <0.001 | 0.51 (0.45-0.58), <0.001 | 0.54 (0.50-0.58), <0.001 | 0.57 (0.50-0.65), <0.001 |
| ADL disability score: 4-6 | 1.00 (ref) | 0.37 (0.31-0.45), <0.001 | 0.41 (0.33-0.50), <0.001 | 0.30 (0.27-0.35), <0.001 | 0.34 (0.26-0.44), <0.001 |
| Age >= 100 years |  |  |  |  |  |
| All participants | 1.00 (ref) | 0.85 (0.75-0.97), 0.018 | 0.67 (0.57-0.78), <0.001 | 0.82 (0.76-0.89), <0.001 | 0.87 (0.77-0.97), 0.015 |
| ADL disability score: 1-3 | 1.00 (ref) | 0.90 (0.77-1.04), 0.147 | 0.73 (0.62-0.88), 0.001 | 0.85 (0.78-0.93), <0.001 | 0.93 (0.82-1.06), 0.261 |
| ADL disability score: 4-6 | 1.00 (ref) | 0.69 (0.54-0.88), 0.003 | 0.45 (0.34-0.61), <0.001 | 0.68 (0.59-0.79), <0.001 | 0.68 (0.56-0.84), <0.001 |
| ^a^ with adjustment for sex, education, marital status, residence, co-residence, current smoking, current drinking, current regular exercise, regular intake of foods, multimorbidities, and self-rated health.  Abbreviations: ADL = activities of daily living, CI = confidence interval, TR = time ratio. | | | | | |

| Table S7. Association between duration of ADL disability and overall survival by multimorbidities | | | | | |
| --- | --- | --- | --- | --- | --- |
| Adjusted TR (95% CI)^a^, p | Non-ADL disability | Duration of ADL disability | | | |
|  |  | Less than 6 months | 6 to 12 months | 12 to 60 months | At least 60 months |
| Without multimorbidities |  |  |  |  |  |
| All participants | 1.00 (ref) | 0.77 (0.70-0.84), <0.001 | 0.65 (0.59-0.72), <0.001 | 0.78 (0.74-0.83), <0.001 | 0.78 (0.71-0.86), <0.001 |
| ADL disability score: 1-3 | 1.00 (ref) | 0.79 (0.71-0.87), <0.001 | 0.69 (0.61-0.78), <0.001 | 0.82 (0.77-0.87), <0.001 | 0.82 (0.74-0.91), <0.001 |
| ADL disability score: 4-6 | 1.00 (ref) | 0.72 (0.61-0.85), <0.001 | 0.52 (0.42-0.65), <0.001 | 0.67 (0.59-0.75), <0.001 | 0.64 (0.52-0.78), <0.001 |
| With multimorbidities |  |  |  |  |  |
| All participants | 1.00 (ref) | 0.66 (0.59-0.74), <0.001 | 0.68 (0.59-0.78), <0.001 | 0.71 (0.66-0.77), <0.001 | 0.85 (0.75-0.97), 0.016 |
| ADL disability score: 1-3 | 1.00 (ref) | 0.72 (0.63-0.82), <0.001 | 0.76 (0.64-0.90), 0.002 | 0.78 (0.72-0.85), <0.001 | 0.95 (0.82-1.11), 0.516 |
| ADL disability score: 4-6 | 1.00 (ref) | 0.45 (0.36-0.57), <0.001 | 0.52 (0.41-0.66), <0.001 | 0.53 (0.47-0.61), <0.001 | 0.63 (0.51-0.79), <0.001 |
| ^a^ with adjustment for sex, age, education, marital status, residence, co-residence, current smoking, current drinking, current regular exercise, regular intake of foods, and self-rated health.  Abbreviations: ADL = activities of daily living, CI = confidence interval, TR = time ratio. | | | | | |

| Table S8. Association between duration of ADL disability and overall survival after excluding deaths within the first year or the first two years | | | | | |
| --- | --- | --- | --- | --- | --- |
| Adjusted TR (95% CI)^a^, p | Non-ADL disability | Duration of ADL disability | | | |
|  |  | Less than 6 months | 6 to 12 months | 12 to 60 months | At least 60 months |
| Within the first year | |  |  |  |  |
| All participants | 1.00 (ref) | 0.79 (0.74-0.85), <0.001 | 0.73 (0.67-0.79), <0.001 | 0.81 (0.78-0.84), <0.001 | 0.84 (0.78-0.90), <0.001 |
| ADL disability score: 1-3 | 1.00 (ref) | 0.82 (0.76-0.88), <0.001 | 0.76 (0.69-0.83), <0.001 | 0.83 (0.80-0.87), <0.001 | 0.88 (0.81-0.95), 0.001 |
| ADL disability score: 4-6 | 1.00 (ref) | 0.70 (0.61-0.80), <0.001 | 0.64 (0.55-0.76), <0.001 | 0.73 (0.67-0.80), <0.001 | 0.70 (0.61-0.81), <0.001 |
| Within the first two years | |  |  |  |  |
| All participants | 1.00 (ref) | 0.86 (0.80-0.92), <0.001 | 0.77 (0.71-0.83), <0.001 | 0.87 (0.84-0.91), <0.001 | 0.85 (0.80-0.91), <0.001 |
| ADL disability score: 1-3 | 1.00 (ref) | 0.88 (0.82-0.95), 0.001 | 0.77 (0.71-0.84), <0.001 | 0.88 (0.85-0.93), <0.001 | 0.88 (0.82-0.94), <0.001 |
| ADL disability score: 4-6 | 1.00 (ref) | 0.77 (0.67-0.88), <0.001 | 0.75 (0.62-0.90), 0.003 | 0.82 (0.74-0.91), <0.001 | 0.74 (0.64-0.86), <0.001 |
| ^a^ with adjustment for sex, age, education, marital status, residence, co-residence, current smoking, current drinking, current regular exercise, regular intake of foods, multimorbidities, and self-rated health.  Abbreviations: ADL = activities of daily living, CI = confidence interval, TR = time ratio. | | | | | |

| Table S9. Association between duration of ADL disability and overall survival, in considering the losses censored at varying time of follow-up | | | | | |
| --- | --- | --- | --- | --- | --- |
| Adjusted TR (95% CI)^a^, p | Non-ADL disability | Duration of ADL disability | | | |
|  |  | Less than 6 months | 6 to 12 months | 12 to 60 months | At least 60 months |
| Considering the losses censored at the median of follow-up |  |  |  |  |  |
| All participants | 1.00 (ref) | 0.80 (0.75-0.86), <0.001 | 0.74 (0.68-0.80), <0.001 | 0.83 (0.79-0.87), <0.001 | 0.89 (0.83-0.96), 0.003 |
| ADL disability score: 1-3 | 1.00 (ref) | 0.84 (0.77-0.90), <0.001 | 0.78 (0.71-0.86), <0.001 | 0.86 (0.82-0.90), <0.001 | 0.91 (0.84-0.99), 0.029 |
| ADL disability score: 4-6 | 1.00 (ref) | 0.73 (0.64-0.83), <0.001 | 0.65 (0.56-0.76), <0.001 | 0.76 (0.70-0.83), <0.001 | 0.87 (0.75-1.00), 0.050 |
| Considering the losses censored at the end of follow-up |  |  |  |  |  |
| All participants | 1.00 (ref) | 0.88 (0.80-0.98), 0.022 | 0.82 (0.72-0.93), 0.002 | 0.90 (0.84-0.97), 0.004 | 0.99 (0.88-1.10), 0.811 |
| ADL disability score: 1-3 | 1.00 (ref) | 0.92 (0.82-1.03), 0.156 | 0.87 (0.75-1.00), 0.053 | 0.90 (0.84-0.97), 0.007 | 0.94 (0.83-1.06), 0.299 |
| ADL disability score: 4-6 | 1.00 (ref) | 0.83 (0.68-1.01), 0.068 | 0.72 (0.57-0.91), 0.005 | 0.96 (0.84-1.09), 0.509 | 1.21 (0.98-1.51), 0.082 |
| ^a^ with adjustment for sex, age, education, marital status, residence, co-residence, current smoking, current drinking, current regular exercise, regular intake of foods, multimorbidities, and self-rated health. Abbreviations: ADL = activities of daily living, CI = confidence interval, TR = time ratio. | | | | | |

| Table S10. Association between duration of ADL disability and overall survival after multiple imputation^a^ | | | | | |
| --- | --- | --- | --- | --- | --- |
| Adjusted TR (95% CI)^b^, p | Non-ADL disability | Duration of ADL disability | | | |
|  |  | Less than 6 months | 6 to 12 months | 12 to 60 months | At least 60 months |
| All participants | 1.00 (ref) | 0.68 (0.64-0.73), <0.001 | 0.67 (0.62-0.72), <0.001 | 0.72 (0.70-0.75), <0.001 | 0.78 (0.73-0.83), <0.001 |
| ADL disability score: 1-3 | 1.00 (ref) | 0.74 (0.69-0.80), <0.001 | 0.72 (0.66-0.78), <0.001 | 0.79 (0.76-0.83), <0.001 | 0.84 (0.78-0.90), <0.001 |
| ADL disability score: 4-6 | 1.00 (ref) | 0.56 (0.50-0.62), <0.001 | 0.56 (0.49-0.64), <0.001 | 0.58 (0.54-0.62), <0.001 | 0.65 (0.59-0.72), <0.001 |
| ^a^ multiple imputation was performed by chained equations to create 10 datasets, of which the resultant model estimates for each were combined using Rubin`s rules (). The present sample size was a little different from the sample size in the flow chart, which was caused by the partial overlap between the lost participants and the participants with missing baseline data.  ^b^ with adjustment for sex, age, education, marital status, residence, co-residence, current smoking, current drinking, current regular exercise, regular intake of foods, multimorbidities, and self-rated health.  Abbreviations: ADL = activities of daily living, CI = confidence interval, TR = time ratio. | | | | | |

Figure S1. The distributions of (A) ADL disability score and (B) duration of ADL disability in participants suffering from ADL disability at baseline.


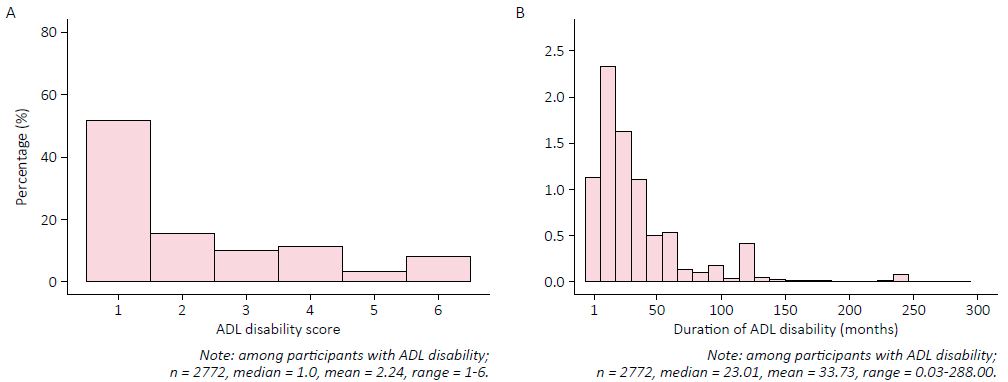


Abbreviations: ADL = activities of daily living.

Figure S2. Kaplan-Meier curves plotting survival probability of non-ADL disability participants and those with ADL disability in different severity.


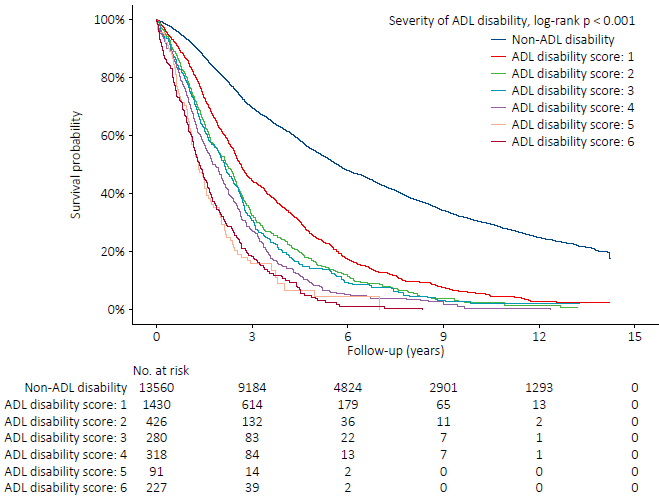


Figure S3. Visualized association between duration of ADL disability and survival in participants with different severity of ADL disability.


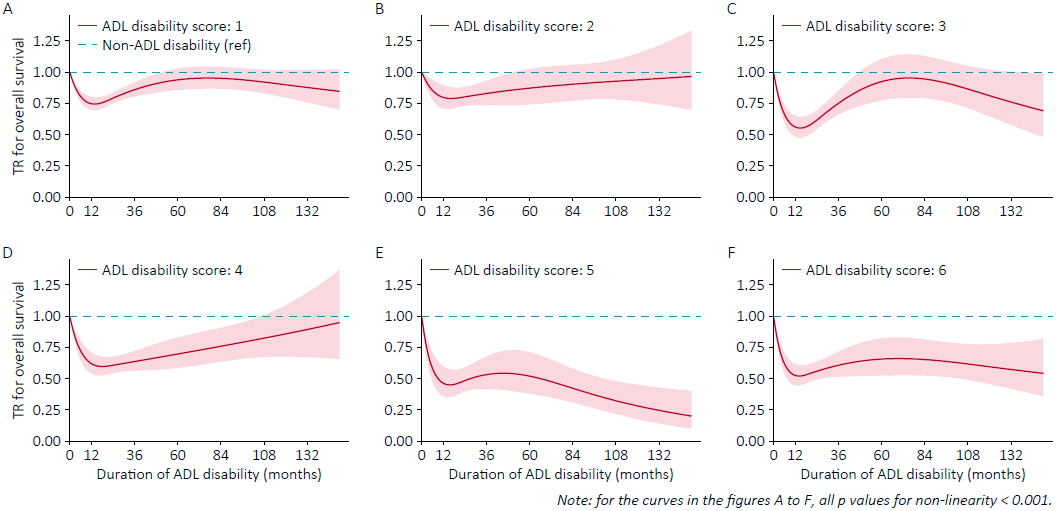


With non-ADL disability as reference, the red solid lines depict the trend in overall survival with longer duration of ADL disability by different score, while shadow represents corresponding 95% CIs. Duration of ADL disability was modeled using a restricted cubic spline with four knots at the 5th, 35th, 65th, and 95th percentiles, and TRs and 95% CIs were derived from AFT model adjusted for sex, age, education, marital status, residence, co-residence, current smoking, current drinking, current regular exercise, regular intake of foods, comorbidities, and self-rated health.

Abbreviations: ADL = activities of daily living, AFT = accelerated failure time, CI = confidence interval, TR = time ratio.
